# Angiosarcoma of the thyroid in an old man

**DOI:** 10.1186/1471-2318-11-S1-A48

**Published:** 2011-08-24

**Authors:** P Petronella, F Freda, M Ferretti, D Fierro, M Scorzelli, S Canonico

**Affiliations:** 1 U.O. of Geriatic Surgery, Department of Gerontology, Geriatry and Metabolic Disease, School of Medicine, Second University of the Study of Naples, Italy

## Background

The angiosarcoma of the thyroid gland is a primary malignant highly aggressive tumor. It is classified as a malignant vascular thyroid lesion of mesenchymal origin. In addition to the neck and head, the majority of angiosarcomas also originate from the skin and soft tissues, or limbs of patients with lymphedema.

Angiosarcoma of the thyroid is rare and the greatest incidence is witnessed near the Alps. It constitutes only 2-10% of malignant thyroid tumors in Switzerland, Austria and Northern Italy.

The prevalence in the Alpine regions can be due probably to iodine deficiency with a long history of endemic goiter. It strikes in old age. There are cases in which the tumor occurs without a history of goiter, and it the occurrence is very unusual in non-alpine areas, so it seemed interesting to present a case involving this type.

Due to the increase of the thyroid volume, often occurring in a short time, dyspnea is a frequent symptom, associated with deviation of the tracheal axis.

From the macroscopic point of view, this tumor typically appears to be with big dimensions, with large areas of necrosis and hemorrhage. Microscopically, freely anastomosed channels are often associated with a papillary configuration having a pattern of predominant intraluminal growth; the nuclei of the epithelioid endothelial cells are large, vesicular, smooth-contoured, with a large basophilic or amphophylic nucleolus connected by chromatin strands to the nuclear membrane. Typical and atypical mitosis were found in large numbers; the growth pattern is usually highly invasive and tumor necrosis is very strong. Of fundamental importance is the peculiarity that tumor cells express vascular markers such as Factor VIII, CD 31 and CD 34.

The distinction between angiosarcoma and anaplastic sarcomatoid carcinoma is difficult and the same expression of the angiosarcoma has been subject to dispute.

Clinical history: most of these tumors appear as a poorly encapsulated and infiltrating mass, which tends to grow in the absence of pain. Local recurrence, even after complete excision, and metastasis are common.

After diagnosis, patients often die quickly. This type of cancer typically metastasizes in the first instance at the level of regional lymph nodes and lung, in the late stages in the bone marrow. Multimodal treatment, a widely accepted approach, envisages surgery, radiotherapy and chemotherapy.

The study was inspired by the observation of a case of thyroid hemangiosarcoma in a 71-year-old man with a history of goiter for twenty years, who decided to undergo an operation for the worsening of the dyspnea.

## Case report

A 71-year-old male came to our department complaining of dyspnea, hypoventilation and dysphonia, determined by swelling in the neck region, related to considerable increase of the thyroid gland.

On examination, the thyroid gland also appeared fairly static during the acts of deglutition. With this pathology, the patient had been living for more than twenty years, without any type of therapy.

An ultrasound examination dating back to 1997, documented a complete subversion of the echotexture of the whole gland and the presence of a large nodule in the right lobe displaying a complex echotexture. A further ecography in the year 2000 documented the increase in volume of the thyroid which was also the cause of the right carotid bulb dislocation.

Among the laboratory tests, we also found significantly elevated thyroglobulin values. The preoperative FNAC was not significant and we decided to not repeat it for the necessary surgical indication due to worsening dyspnea.

Neck and chest CT was performed without contrast from which was noticed, in the right lobe of the thyroid, a voluminous expansive formation characterized by a hyperdense rim surrounding large areas of liquid hypodensity and the presence of lymph node nodules in the right submandibular region. Also the left lobe of the thyroid appeared increased in size with hypodense nodular formations of colloid cyst type. It also showed a small nodular formation, of about 3mm in size, of dubious diagnostic ascription, in the posterior basal segment of the right lower lobe of the lung.

Although surgical operation was expected to be very complex, it was performed in a completely linear way. The left lobe was easily separable from the surrounding tissue; it appeared in the throes of a nodular transformation and was immersed. The right lobe appeared uniformly in the throes of nodular and colloid cyst transformation and penetrated the upper part of the neck, adhering to the vessels, from which, however, it was easily dissociated; it measured about 15 cm. Lymph nodes were not visible. Surgical times were also lower than expected, particularly the removal of the right half was relatively easy and the mass appeared well encapsulated and demarcated.

Histo-citopathological analysis revealed for the right lobe a nodule composed of necrotic hemorrhagic areas surrounded by a fibrous capsule within which there were anastomosing channels bordered by atypical epithelioid cells. The positivity for CD31 and factor VIII and the negativity for CK deposed for the diagnosis of thyroid angiosarcoma.

## Discussion

Our case’s peculiarities are represented by the patient’s lack of provenance from an Alpine area and by the absence of rapid growth, behavior that diverges from most of the cases described in literature. Diagnosing the angiosarcoma is not easy even on a histological examination, and it must be supported by detailed investigations (positivity for CD31 and factor VIII, negativity for CK). Immunohistochemical markers of endothelial differentiation and the demonstration of Weibel-Palade bodies at ultrastructural level support the idea that the origin of this tumor is endothelial. Cytocheratine expression and morphological mimicry of epithelioid angiosarcoma with anaplastic carcinoma highlight the importance of using a broad panel of immunostains in the diagnostic workup of these tumors, in conjunction with a careful microscopic and ultrastructural study. CD31 is considered the most sensitive and specific marker for endothelial differentiation, being expressed in 90% of angiosarcomas and in slightly more than 1% of carcinomas; the endothelial marker CD34 and the antigen related to factor VIII should be added to the panel too.

A hypothesis that derives from our observation concerns the role of thyroglobulin testing; there are not many studies that evaluate the correlation with the angiosarcoma, so further work can be envisaged.

In conclusion, we must bear in mind the possibility of the existence of thyroid angiosarcoma in the presence of long-standing goiter, especially if there has been rapid growth. We report and confirm the diagnosis as even the anatomo-pathologist examination may encounter some difficulties in distinguishing it from anaplastic carcinoma. Therefore, it is essential to search for markers such as CD31, CD34 and factor VIII. Finally, we re-emphasize that the growth is not always extremely rapid, as in our case.

**Figure 1:**
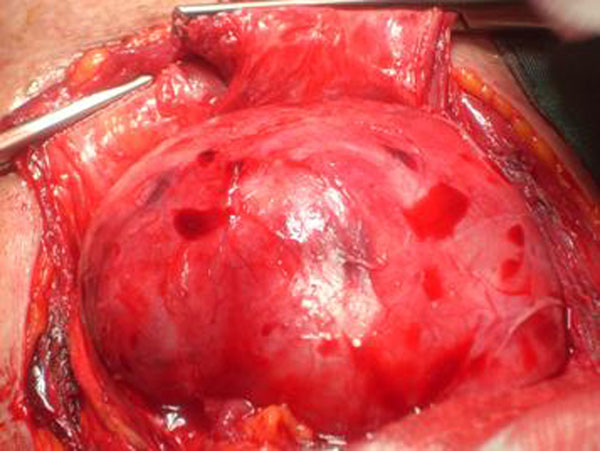
The thyroid during surgery

**Figure 2:**
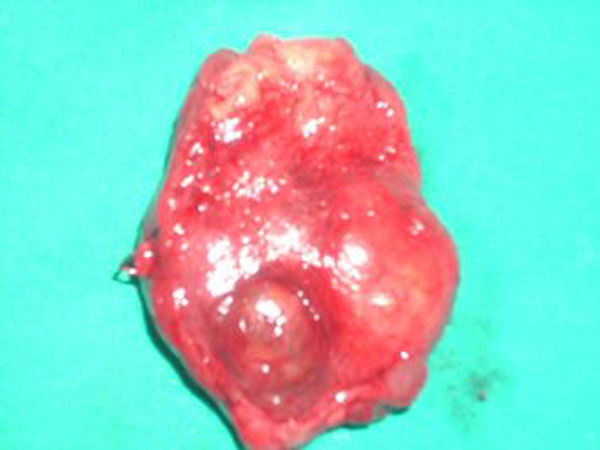
Left lobe and isthmus of the thyroid

**Figure 3:**
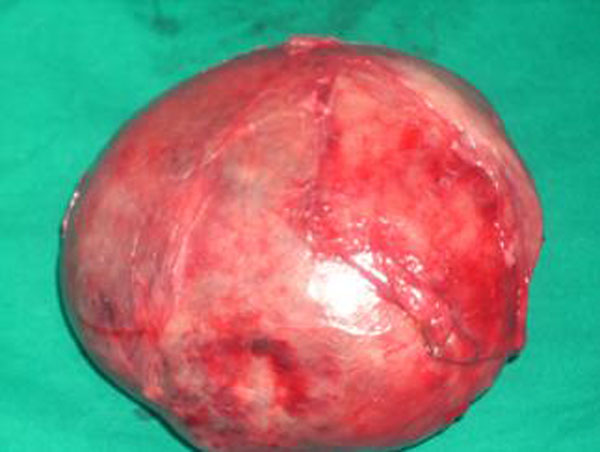
Right lobe of the thyroid
